# Multi-omic integration with human DRG proteomics highlights TNFα signalling as a relevant sexually dimorphic pathway

**DOI:** 10.1101/2024.12.06.626968

**Published:** 2024-12-10

**Authors:** Allison M Barry, Julia R Sondermann, Joseph B Lesnak, Feng Xian, Úrzula Franco-Enzástiga, Jayden A O’Brien, David Gomez Varela, Morgan K Schackmuth, Stephanie Shiers, Theodore J Price, Manuela Schmidt

**Affiliations:** 1 Department of Neuroscience and Center for Advanced Pain Studies, University of Texas at Dallas, Richardson, TX 75080, USA; 2 Systems Biology of Pain, Division of Pharmacology & Toxicology, Department of Pharmaceutical Sciences, University of Vienna, Austria

## Abstract

The peripheral nervous system has been widely implicated in pathological conditions that exhibit distinct clinical presentations in men and women, most notably in chronic pain disorders. Here, we explored this sexual dimorphism at a molecular level. We expanded the available omics landscape in the PNS to include quantitative proteomics of the human dorsal root ganglia (hDRG) and nerve. Using data-independent acquisition mass spectrometry, we uncovered an extensive protein landscape, validated against tissue-specific differences between the nerve and hDRG. Using a combination of multi-omic analyses and *in vitro* functional support, we then examined sex-differences, highlighting TNFα signalling as a relevant sexually dimorphic pathway in males. These results support a functional sexually dimorphism in the periphery, which is of particular importance to sensory- and pain-related clinical translation.

## Introduction

Neuroimmune- and pain- related condition prevalence, as well as the corresponding treatment efficacy, can differ across sex and gender (Greenspan et al., 2007; Mogil, 2012). Understanding sexual dimorphism at a molecular level is thus a fundamental clinical issue. Autoimmune conditions, for example, are highly biased toward women (upwards of 80%), and infections can elicit differing immune responses. Women also have heightened sensitivity to pain in an experimental context, show higher rates of chronic pain, and are the dominant gender in many pain-centric disorders, from migraine to complex regional pain syndrome (CRPS) (Berkley, 1997; Dawes & Bennett, 2021; Mogil, 2012).

Pre-clinical work in rodents shows a similar sexual dimorphic trend at the immune and nervous system level. Decades of work suggest complex, cross-species mechanism(s) underlying these clinical presentations. Hormones such as oestrogen, prolactin, and testosterone have been implicated in a range of pain and neuro-immune conditions, while evidence for central and peripheral sexually dimorphic mechanisms have been shown (Ikegami et al., 2022; Ray et al., 2023; Sorge et al., 2011, 2015; Yu et al., 2020).

In the somatosensory system, differences are commonly discussed in the peripheral nervous system. Neuronal afferents project from the skin and viscera to the dorsal horn of the spinal cord, with cell bodies in the dorsal root ganglia (DRG, in humans - hDRG). The DRG contain sensory neuron cell bodies, as well as a diverse set of non-neuronal cells. These sensory neurons respond to inflammation by detecting immune mediators and then releasing neuropeptides in complex neuro-immune circuits. At a molecular level, transcriptional profiles of these cell types have recently been published for human sensory neurons (Bhuiyan et al., 2024; Tavares-Ferreira et al., 2022), as well as non-neuronal cells (Bhuiyan et al., 2024; Jung et al., 2023) with some differences in gene expression reported across male and female donors. In bulk tissue, recent molecular profiling of the hDRG has highlighted differentially accessible chromatin regions (DARs), as well as sex-specific changes in neuropathic pain states at the transcriptome level (Franco-Enzástiga et al., 2024; Ray et al., 2023). To date, the corresponding proteome signature has been missing, as prior studies depend on shotgun (data dependent acquisition) proteomics, a useful technique but biased towards high-abundant proteins (Doty et al., 2022; Schwaid et al., 2018).

Here, we generate a quantitative hDRG proteomic dataset across male and female donors. This comprehensive dataset provides a human-centric reference for protein expression in a key structure of the peripheral nervous system, and complements previously published ATAC-seq and RNA-seq data. Through integration across omics from the same tissue, we show strong evidence for sexual dimorphism in the TNFα signalling pathway. Genome-wide association studies (GWAS) provide support that it is a functionally relevant pathway in the periphery, and evidence from clinical trials highlights a link to clinical sexual dimorphism in response to medication against these targets. The functional relevance of TNFα signalling in the hDRG was confirmed *in vitro*, and downstream changes in protein phosphorylation suggests a possible mechanism of action. Together, these findings speak to a sexually dimorphic pathway in the peripheral nervous system, which is of particular importance to sensory- and pain-related translation.

## Results

**Figure 1.**
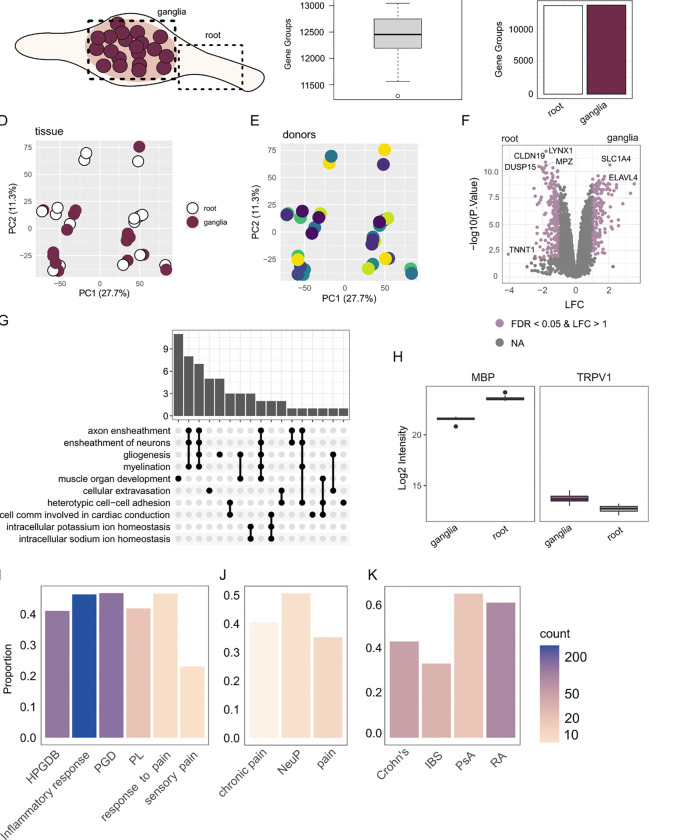
Quantitative proteomics of the hDRG and nerve root. A. Schematic of the ganglia and nerve root (NR, root). B. Gene Groups (protein counts) per sample. C. Gene Group count per tissue. D-E: PCA for tissue (D) and donor ( E). F. Volcano plot of differentially expressed proteins (DEPs, ganglia vs nerve root; positive LFC for ganglia), coloured by abs(LFC) and FDR < 0.05. G. Upset plot showing GO term over enrichment analysis for differentially expressed proteins upregulated in the nerve root. H. Example DEPs for myelin basic protein (MBP) and TRPV1 (padj < 0.01 each). I-K. Overlap of detected Gene Groups with relevant gene sets: related to pain (I), pain drugs (J), peripheral autoimmune conditions (K). HPGDB: Human Pain Genetics Database (Meloto et al., 2018), PGD: Pain Gene Database (LaCroix-Fralish et al., 2007), PL: DoloRisk priority group Pain List (Themistocleous et al., 2023). NeuP: neuropathic pain. IBS: Irritable Bowel Syndrome, PsA: Psoriatic Arthritis, RA: Rheumatoid Arthritis. Related to SFig 1.

In this study, we present - to our knowledge - the first quantitative proteomic dataset of the human dorsal root ganglia (hDRG) and nerve root (Fig 1A). Using data independent acquisition coupled with parallel accumulation serial fragmentation (DIA-PASEF) we identify ~ 12500 Gene Groups (proteins) per sample, with similar levels between the ganglia and root (Fig 1B–C, see Data Availability). Donor details are available in Table 1. We confirmed data quality using control samples throughout the MS runs for both LC-MS and SP3 replicates (SFig 1A), and do not see overt variance by donor or age (SFig 1B). As expected, samples show the highest correlation within sample replicates (SFig 1C). Even so, samples do not cluster obviously by tissue type or donor (Fig 1D–E) or by sex/replicate (SFig 1D), suggesting a generalized proteome signature across all samples. These replicates were merged (by mean) for all downstream analyses.

When comparing the nerve root to the ganglia, we see large differences in protein (aka “Gene Group”; see methods) levels. These differentially expressed proteins (DEPs) show a bias in myelin-associated terms upregulated in the root and soma-related terms in the ganglia (Fig 1F–H). A full list of DEPs is provided in Supplemental Table 1, with GO enrichment in Supplemental Tables 2–3. This is expected, given our knowledge of these tissues, and gives confidence to this dataset to probe other questions. With our current dataset quality and coverage, we see this data as a reference for the hDRG proteome, with extensive coverage of relevant curated gene lists for pain targets (Fig 1I), pain-related drug targets (Fig 1J), and peripheral autoimmune targets (Fig 1K).

**Figure 2.**
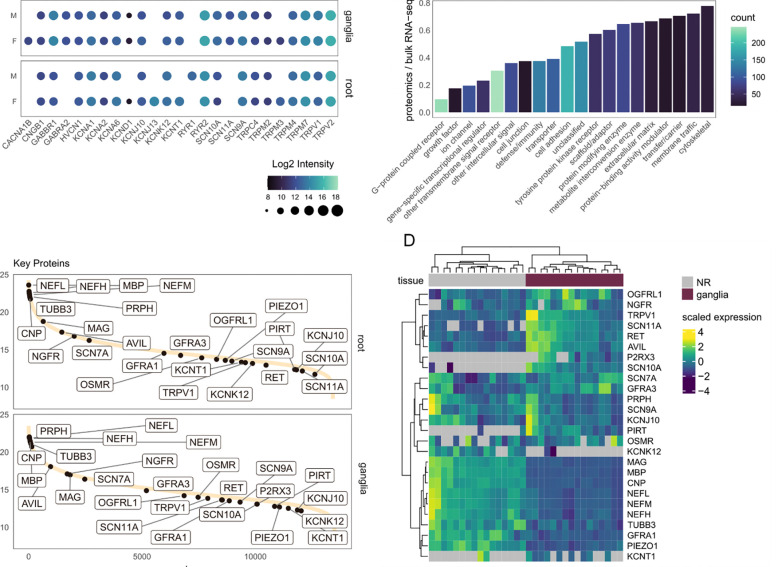
Ion channel and G-protein coupled receptor (GPCR) expression across tissues. A. Average expression by ion channel across tissues. B. Proportion of receptor detection in the proteomics data vs bulk RNA-seq of the hDRG (Ray et al., 2023). C. Dynamic range plot showing ranked intensity across tissues. Key proteins (gene groups) for sensory neurons and myelin are highlighted. D. Heatmap across all samples (including each replicate) highlighting the dataset completeness for key proteins in C.

Given the importance of membrane proteins in neurological and neuro-immune conditions in the PNS, as well as the previous difficulties in detecting membrane proteins using mass spectrometry, we specifically investigated ion channel and receptor abundance in our dataset (Fig 2) (Schwaid et al., 2018). Here, we detect a number of TRP-, SCN-, and KCN- ion channels (Fig. 2A), among others, as well as a small number of GPCRs (Fig 2B). These candidates are expressed throughout our dynamic range of protein abundance (Fig 2C), with a number of key proteins detected almost exclusively in the ganglia (eg. P2RX3, SCN10A, Fig 2D). Additionally, when we compare these data to a curated list of proteins for sensory neurons and myelin, we see a clear separation of tissue types, again highlighting the strength of this dataset (Fig 2D).

### The Ganglia and Nerve Root

**Figure 3.**
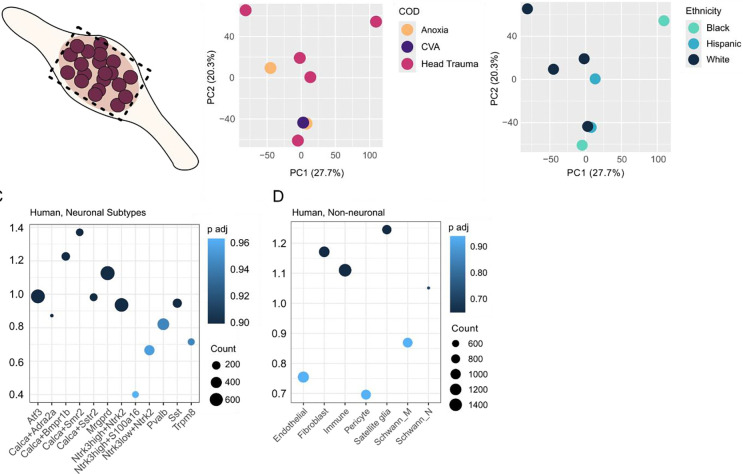
DIA-PASEF proteomics of the hDRG has markers across cell types. A. Schematic. B. PCA, coloured by cause of death (COD, left) and Ethnicity (right). C. GSEA human neuronal subtype markers. D. GSEA against non-neuronal cell type markers. Related to SFig. 2–3.

Because of the differences between the ganglia and nerve root (Fig 1F–H), we independently investigated the proteome of each tissue type. In the ganglia (Fig 3A), we again do not see obvious clustering by PCA over cause of death (COD) and Ethnicity (Fig 3B–C). The samples cluster by replicate (SFig 2A) and show consistent protein counts (SFig 2B). In line with previous reports for quantitative proteomics, the data also does not correlate strongly to bulk RNA-seq of the same tissue (Ray et al., 2023), with an R^2^ = 0.15 (SFig 2C). When looking more precisely at receptor types, we see a highly variable correlation across receptor types, ranging from −0.2 to 0.8 (SFig 2D), likely reflecting a combination of biological and technical differences in receptor type proteomics, due in part to regulatory dynamics.

Next, we examined the enrichment profiles of neuronal and non-neuronal cells using custom genesets derived from snRNA-seq (Bhuiyan et al., 2024) (Fig 3D, see: methods). Here, we do not see a specific significant enrichment for any cell type, but instead can detect marker genes across populations. This trend is mirrored when using gene sets derived from mouse datasets (Zheng et al., 2019) (SFig 3A–B), and for neurons using a secondary, visium-based, hDRG dataset (Tavares-Ferreira et al., 2022) (SFig 3C).

**Figure 4.**
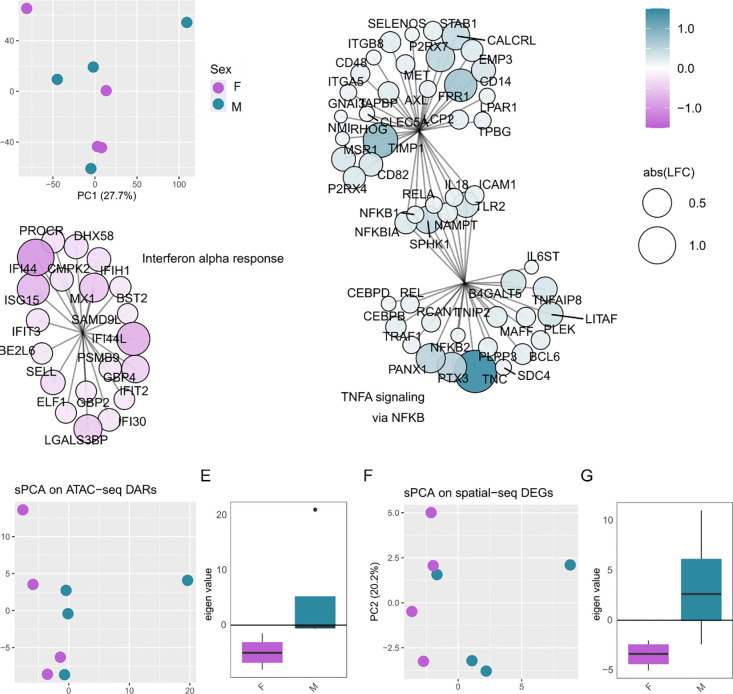
Sexual dimorphism in the hDRG. A. PCA of ganglia samples (merged by replicate). B-C. Representative GSEA pathway enrichments (male = positive, female = negative LFC). B. TNFα signalling via NFκB with overlap to Inflammatory response. C. IFNα response. Supervised PCA (sPCA) and corresponding eigen values (PC1) of ganglia samples on DARs (ATAC-seq, (Franco-Enzástiga et al., 2024)) (D-E), and DEGs (RNA-seq, (Tavares-Ferreira et al., 2022)). Related to SFig 4–7.

We then set out to investigate male/female differences in the hDRG (Fig 4, SFig 4). Ganglia samples do not cluster by sex (Fig 4A) and no differentially expressed proteins were detected between males and females (SFig 4A, Supplemental Table 4–5). Even so, GSEA enrichment highlights pathway differences (Fig 4B–C, SFig 4B–D). Notably, TNFα signalling is enriched in male donors (Fig 4B), while interferon (IFN) α response (Fig 4C) and Oxidative Phosphorylation (SFig 4D) are enriched in female samples.

To see if there was a shared signature of sexual dimorphism across omics types (ie. ATAC-seq and RNA-seq), we next clustered the ganglia samples by supervised PCA (sPCA) against previously published DARS (Franco-Enzástiga et al., 2024) and differentially expressed genes (DEGs, (Tavares-Ferreira et al., 2022)) comparing hDRG of male and female donors (Fig 4D–G).

We see a separation of male and female proteomic samples by sPCA against sexually dimorphic DARs (ATAC-seq, Fig 4D–E) and DEGs (RNA-seq, Fig 4F–G), although this trend is not statistically significant (Welch’s, p ~ 0.1) in either case. Eigengenes were extracted to see what was driving this separation, with a number of proteins in the TNFα signalling pathway showing high loading values, suggesting they contribute to driving the difference across sex (SFig 5).

To see if the pathway changes from our proteomic data are shared across omics, we next performed GSEA against pseudobulk spatial ATAC-seq (SFig 6A), as well as bulk RNA-seq from thoracic vertebrectomy participants (SFig 6B) (Franco-Enzástiga et al., 2024; Ray et al., 2023). TNFα signalling via NFκB was enriched in males across datasets, suggesting a cross-omic pattern of sexual dimorphism.

The nerve root (SFig 7A) exhibits slightly fewer protein identifications (SFig 7B) than the ganglia, but again do not cluster by sex (SFig 7C) or show differentially expressed proteins by sex (SFig 7D, Supplemental Table 6–7). Mirroring the GSEA from Fig 4, we see a different pattern of sexual dimorphism in the nerve root compared to the ganglia. Like the ganglia, we see an enrichment for Oxidative Phosphorylation in female donors, but do not replicate the differences in IFNα Response or TNFα signalling via NFκB (SFig 7E). By sPCA, we also do not see a clear separation by sex using DARs (SFig 7F) or DEGs (SFig 7G) from the corresponding hDRG ATAC-seq and RNA-seq datasets (Franco-Enzástiga et al., 2024; Tavares-Ferreira et al., 2022).

### Sexual dimorphism across omics datasets

**Figure 5.**
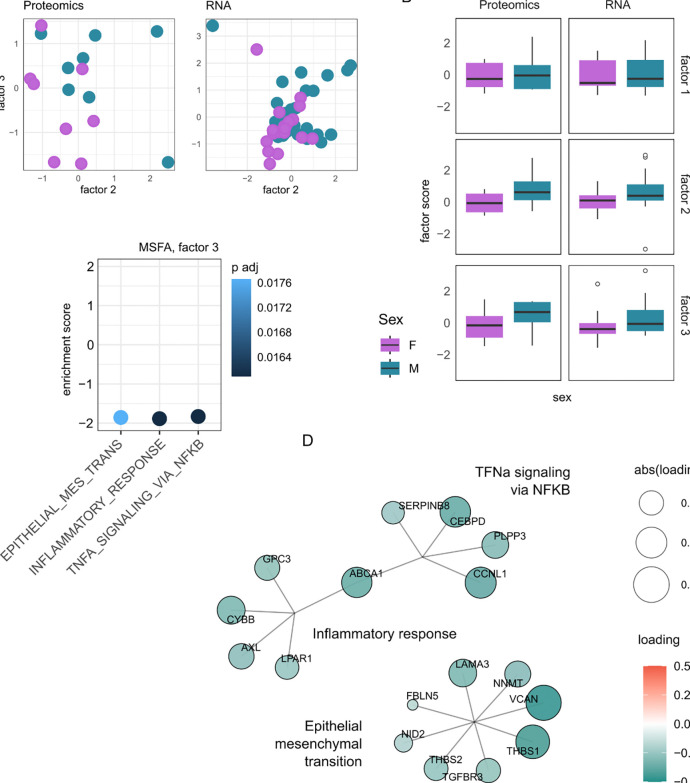
Multi-study factor analysis (MSFA) between bulk RNA-seq and bulk proteomic data, based on differentially accessible regions from bulk ATAC-seq. A. Scatter plot by factor, for proteomics (left) and transcriptomics (right). B. Boxplot of Factor scores, stratified by sex, bars = ±1.5*interquartile range. C. GSEA on factor 3 (ranked loadings). D. Pathways + gene loadings from GSEA output of Factor 3. Related to SFig 8.

To probe shared patterns of sexual dimorphism across omics types, we used a multi-study factor analysis (MSFA) on available hDRG datasets (Fig 5, SFig 8) (Franco-Enzástiga et al., 2024; Ray et al., 2023). MSFA allows us to examine class separation (here, male and female) through shared factors across omics types, where each relevant factor has interpretable underlying constructs.

We used the most comprehensive bulk RNA-seq dataset available to date, looking at neuropathic pain in a participant cohort of thoracic vertebrectomy patients undergoing surgery. Of the 70 DRG sequenced, 50 showed neuronal enrichment, as described in the original publication (Ray et al., 2023). These 50 DRG, removed during thoracic vertebrectomy surgical procedure, were stratified by sex for comparison to our hDRG proteomic dataset (both ganglia and nerve root). There were too few samples from the hDRG ATAC-seq datasets for a factor analysis, thus we instead restricted our factor analysis to gene/proteins which correspond to DARs between male and female donors (see methodology) (Franco-Enzástiga et al., 2024).

Likely because of the small sample sizes, only 3 shared factors were extracted, with Factors 2–3 both showing a separation by sex (Fig 5A–B). GSEA against the ranked loading revealed no hallmark pathway enrichment for Factor 2, but 3 enriched pathways for Factor 3: TNFα signalling via NFκB, Inflammatory Response, and Epithelial Mesenchymal Transition (Fig 5C–D, SFig 8A). While there are overlapping terms driving TNFα signalling via NFκB and Inflammatory Response, the enrichment of Epithelial Mesenchymal Transition is independent of this (Fig 5D), but was also detected in our proteomics data (SFig 4B), and in bulk RNA-seq (SFig 6B).

### TNFα release from hDRG immune cells

TNFα signalling consistently appears as a sexually dimorphic pathway in these omics datasets, spanning multiple sets of donors and a large cohort of surgical participants.

If the sexual dimorphism we see is functional relevant, we would expect a few things: Firstly, drugs targeting the pathway should show response differences in men and women (they do, Table 2). Secondly, SNPs against core genes in the pathway would be related to pain and sensory processing (they are, Table 3). Here, sexual dimorphism at the genetic level is also discussed for the TNFα promotor in the context of migraine Fawzi et al., (2015).

**Figure 6.**
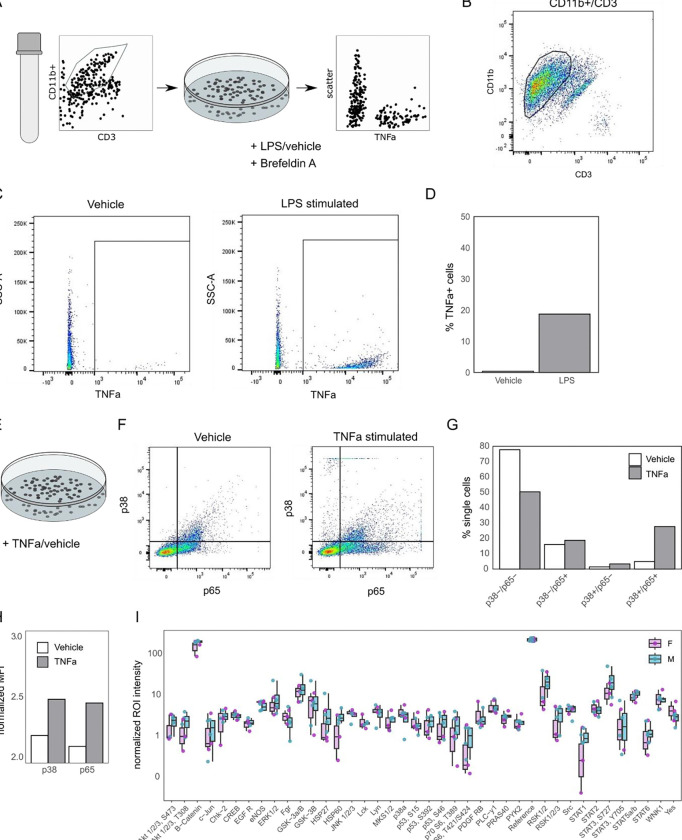
TNFα signalling in the hDRG. A. FACS experiment overview, for isolation and culturing of myeloid cells followed by stimulation *in vitro* (n = 1 donor, Male). B. Myeloid cell gating strategy, full strategy shown in SFigure 9. C. Representative flow cytometry for Vehicle (left) and LPS (right) stimulation of myeloid cells. D. Percentage of TNFα+ single cells after LPS stimulation. E. Schematic for TNFα stimulation of myeloid cells. F. Transcription factor phosphorylation (p38 and p65) from Vehicle (left) and TNFα (right) treated cells. G. Quantification of (F), % of single cells per condition/gate. H. Normalized Mean Fluorescent Intensity (MFI) for p38 and p65 per condition. I. Phosphorylation array of Male (M) and Female (F) hDRG protein samples (anova: Sex (p = 0.0266), Sex:Site interaction (p = 2e-06), n = 4 per group). Related to SFigure 9–10.

To further support the relevance of TNFα sexual dimorphism in the peripheral nervous system, we investigated TNFα release and signalling in the hDRG directly (Fig 6).

To determine if myeloid cells from DRG parenchyma could be a local source of TNFα, we isolated CD11b+ cells from hDRG via FACS (Fig 6A–B). Following isolation, cells were stimulated with LPS (10 ng/mL) or vehicle (RPMI media) along with the intracellular protein transport inhibitor Brefeldin A for 16 hours. Cells were then collected, fixed, permeabilized, and stained for intracellular TNFα production with a TNFα antibody (Fig 6C–D). Following stimulation, there was a higher percentage of cells expressing TNFα staining in the LPS treated (18.7%) when compared to vehicle treated cells (0.36%) (Fig 6C–D), suggesting hDRG myeloid cells could be a local source of TNFα production.

### Phosphorylation in the hDRG

In parallel, we examined if TNFα stimulation could drive increases in the phosphorylation of transcription factors p38 and p65. CD11b+ cells were stimulated with TNFα (10ng/mL) or its vehicle (1X PBS) for 30 minutes (Fig 6E). Cells were then collected, fixed, permeabilized, and stained with p38 and p65 antibodies. Following stimulation there was a higher percentage of CD11b+ cells with staining for p38 and p65 when compared with vehicle treated cells (Fig 6F–G). TNFα treated cells also had a higher median fluorescent intensity signal of both p38 and p65 antibodies (Fig 6H), together suggesting that TNFα can drive increases in the phosphorylation of p38 and p65 in the DRG.

To investigate whether phosphorylation may also be sexually dimorphic (indirectly of TNFα stimulation), we used matched protein samples from our proteomic experiment (4M + 4F) to query sex-specific phosphorylation differences in our pain-free donors (Fig 6I). We see significant differences in Sex (p = 0.0266), and the Sex:Site interaction (p = 2e-06), but we do not see differences for individual phosphorylation sites (Tukey’s HSD, padj ~ 1 for all sites).

## Discussion

This study explores sexual dimorphism in a proteomics dataset of the hDRG. It complements previous ATAC-seq and RNA-seq work, and with deep coverage spanning over 12500 Gene Groups (and ~ 9000 unique proteins) per tissue it provides the most comprehensive proteomic data to date for the hDRG. We report distinct sex differences in small sample sizes with a particular focus on TNFα signalling, highlighting that these differences are seen consistently across cohorts and omics technologies. We have paired this to *in vitro* work on freshly isolated hDRG, confirming the functional relevance of this pathway in the hDRG. Together, this suggests a wide-ranging sexual dimorphism in the hDRG that likely has functional and clinical implications.

In addition to providing the first human-centric DIA-MS proteomics reference of the hDRG, we also improve the coverage over pre-clinical DRG proteomes in terms of ion channel and membrane protein detection (Barry et al., 2018). In the current study, we highlight membrane proteins relevant to pain- and neuroimmune- conditions, even detecting large membrane proteins like PIEZO1.

We validated this dataset across the nerve root and ganglia, highlighting differences in ion channel expression and myelin-related proteins by tissue. In the nerve root, we see an enrichment of terms related to myelination via `myelination`, àxon ensheathment`, and `gliogenesis`, among others, while also observing an enrichment in ion regulation, including `potassium ion homeostasis`, ìntracellular sodium ion homeostasis`, and `sodium ion transmembrane transport`. Fewer GO terms are enriched in the ganglia, these are related to `tRNA aminoacylation` and àmino acid activation` highlighting the role of cell bodies in translation.

When probing sexual dimorphism in the ganglia and nerve root, we see a shared pattern of Òxidative Phosphorylation` enrichment in female donors, as well as differences in `TNFα` and ÌFNα` signalling. Notably, we see TNFα signalling enriched in male ganglia across datasets, spanning from different accessible gene regions (via ATAC-seq, (Franco-Enzástiga et al., 2024)) to different transcriptional profiles in human participants (Ray et al., 2023). By detecting this sexual dimorphism across omics types, as well as across samples from organ donors and human participants, we gain confidence in this being a true biological signal. Paired to this, there is strong evidence from clinical trials that TNFα inhibitors affect men and women differently (Cignarella et al., 2023), suggesting a meaningful molecular signature.

In mice, TNFα signalling in the DRG was recently shown to be sexually dimorphic in an experimental autoimmune encephalomyelitis model (Maguire et al., 2022). In humans, this pathway is frequently studied in circulating immune cells, and TNFα signalling here can be regulated by testosterone levels (Lakshmikanth et al., 2024). Using FACS/flow cytometry on freshly isolated hDRG, we first examined the functional relevance of this pathway in the hDRG specifically: When stimulated, CD11b+ cells from hDRG release TNFα, and TNFα stimulation can alter phosphorylation levels.

Using a targeted phosphorylation approach, we then investigated sexual dimorphism at the level of post-translational modifications. Here, we see an interaction of sex and phosphorylation site in the hDRG. Together this provides an initial avenue for follow-up in humans, as previous work in mice by Maguire and colleagues report phosphorylation differences in the context of the TNFα signalling pathway dimorphism (Maguire et al., 2022).

## Limitations

This dataset presents the highest proteome coverage of the hDRG to date and overcomes many of the known difficulties in detecting membrane proteins, but is not complete. We observe ~20% of ion channels with detectable transcripts in bulk RNA-seq data, and we detect less than 10% of the corresponding GPCRs detected in a comparable bulk RNA-seq dataset (Ray et al., 2023). Prior studies highlight functional expression of membrane proteins like ASICs, opioid receptors (OPRs), and PIEZO2 in DRG which are lacking here, thus the lack of detection is likely technical (given their low abundance), opposed to biological. Increases in sample sizes, paired to further improvements in sensitivity and sample preparation protocols will improve the detection of very low abundant membrane proteins in complex protein mixtures in future work (Rabilloud, 2009; C. C. Wu & Yates, 2003).

## Future Directions

The full implications of this sexual dimorphism remain unclear: previous work in healthy trans men show that taking testosterone (as hormone replacement therapy, HRT) can shift immune function in plasma samples in line with the dimorphism of the hDRG we report here (Lakshmikanth et al., 2024). Within 12 months of starting HRT, these men show increased TNF signalling through NFκB, paired to a decrease in IFNα response, highlighting adaptations to immune function in response to increasing testosterone levels.

Beyond TNFα signalling, varying levels of testosterone in cis men correlate to immune response after vaccination (Furman et al., 2014), and HRT in post-menopausal women can elicit immune changes as early as 1 month (Kamada et al., 2000). Hormone differences are highly relevant in autoimmune conditions as well: while HRT in menopausal women may increase the risk of late-onset RA, hormonal oral contraceptives have been shown to reduce the risk of RA in women (Hadizadeh et al., 2024).

Given the variation across humans, and the adaptive nature of the immune system, the differences reported likely exist on a spectrum which can change with medication and age. In the hDRG specifically, the functional implications require more research. We show that TNFα is released from myeloid cells from DRG parenchyma, and that TNFα stimulation of hDRG immune cells can result in protein phosphorylation, but a direct causation to sexually dimorphic PTMs - and any downstream functional changes - is still needed.

Moving forward, differing immune signatures in their local environment should be accounted for in future studies, especially when considering developmental markers like puberty and menopause.

## Conclusions

This comprehensive, human-centric proteomic dataset complements previously published ATAC-seq and RNA-seq data, providing a quantitative reference proteome of the hDRG. Data are searchable at https://sensoryomics.com/.

Using tissue from both human participants and donors, we show evidence for sexual dimorphism in TNFα signalling spanning the epigenetic signature to proteomic differences. Together, these findings speak to relevant sexually dimorphism in the hDRG, which is of particular importance to sensory- and pain-related translation.

## Methods

### DRG tissue procurement

All human tissue procurement procedures were approved by the Institutional Review Board at the University of Texas at Dallas. Through collaboration with the Southwest Transplant Alliance, human lumbar DRGs (hDRGs, L1-L4) from organ donors were obtained within 4 hours of cross-clamp. Tissue for mass spectrometry was frozen in dry ice right immediately and stored in a −80 °C freezer. All transport was done on large amounts of dry ice to protect tissue integrity. Age-matched male and female samples of mixed ethnicity were used for the current study. Donors were negative for pain, neuropathy, and illicit drug use (excluding marijuana), based on recorded patient history.

For Fluorescent Activated Cell Sorting (FACS) hDRGs were recovered and stored in 10 mL of Hibernate A (BrainBits, HACA500) supplemented with 1% N-2 (Thermo Scientific, 17502048), 2% NeuroCult SM1 (Stemcell technologies, 05711), 1% penicillin/streptomycin (Thermo Fisher Scientific, 15070063), 1% Glutamax (Thermo Scientific, 35050061), 2mM Sodium Pyruvate (Gibco, 11360–070), and 0.1% Bovine Serum Albumin (Biopharm, 71–040) at 4°C until ready for processing (10–16 hrs, see below).

### DRG tissue preparation for mass spectrometry

DRGs were processed sequentially, with experimenters blinded to a randomized order (corresponding to donor column, Table 1). 12–24 hours prior to dissection, tissue was transferred to −20°C. For each hDRG, tissue was thawed on ice for ~15 min prior to dissection (on ice) to remove the surrounding connective tissue. In each ganglia, a region of nerve tissue extending from the core ganglia was identified. This was presumed to contain fewer/no neuronal soma and was transected crudely with a scalpel for separate processing (as the “nerve root” region).

### Protein extraction and SP3-assisted digestion

Due to the size of the ganglia, each ganglia was cut into up to 4 pieces and subsequently, each piece was cut in smaller pieces to increase surface area for lysis. All small pieces of one big cut were transferred to a 2ml LoBind Protein Eppendorf tube (Eppendorf, Hamburg, Germany) prefilled with lysis buffer (2% SDS, 100mM Tris, 5% glycerol, 10mM DTT and 1x protease inhibitor cocktail) and sonicated for 15 min (cycles of 30 sec ON and 30 sec OFF, 4°C, low frequency) in a Bioruptor Pico (Diagenode, Seraing, Belgium). Samples were subsequently incubated for 15 min at 70°C, 1500 rpm in a ThermoMixer C (Eppendorf) followed by a centrifugation for 5 min with 10000 × g at room temperature (RT) to pellet cell debris. The supernatant was mixed with 5x sample volume 100% acetone (pre-chilled at −20°C) and incubated for 2.15–2.45 h to precipitate the proteins.

Precipitates were pelleted by centrifugation for 30 min at 14000 × g, RT. Pellets were washed with ice-cold 80% EtOH and centrifugated again with above mentioned parameters. The EtOH was removed and pellets air-dried for around 20 min at RT. Resolubilization in lysis buffer was facilitated by incubation for 10 min at 70°C, 1500 rpm. Total protein concentration was determined at 280 nm with a NanoPhotometer N60 (Implen, Munich, Germany). Subsequently, samples were flash-frozen and stored at −20°C until further usage.

Protein clean-up and digest was then performed as described in Xian et al., 2022. This is based on the single-pot, solid-phase-enhanced sample preparation (SP3) method from Hughes et al. (Hughes et al., 2019), and is also detailed in the corresponding protocol at protocol.io (Barry et al., n.d.). Remaining protein was used for a targeted phosphorylation array (below).

### Tandem mass spectrometry

Nanoflow reversed-phase liquid chromatography (Nano-RPLC) was performed on NanoElute2 systems (Bruker Daltonik, Bremen, Germany). This was coupled with timsTOF HT (Bruker Daltonik, Bremen, Germany) via CaptiveSpray ion source. Mobile phase A consisted of 100% water, 0.1% formic acid (FA) and mobile phase B of 100% acetonitrile (ACN), 0.1% FA.

500 ng of peptides were loaded onto a C18 trap column (1 mm x 5 mm, ThermoFisher) and further separated over a 90 min gradient on an AuroraTM ULTIMATE column (25 cm × 75 μm) packed with 1.6 μm C18 particles (IonOpticks, Fitzroy, Australia). The flow rate was set to 250 nL/min, except for the last 7 minutes, where the flow rate was accelerated to 400 nL/min. The mobile phase B was linearly increased from 2 to 20% in the first 60 minutes, followed by another linear increase to 35% within 22 minutes and a steep increase to 85% in 0.5 min. Then, a flow rate switch to 400 nL/min was achieved in 0.5 min and was maintained for 7 minutes to the end of the gradient to elute all hydrophobic peptides.

The samples were analyzed in data-independent acquisition (DIA) mode coupled with parallel accumulation serial fragmentation (PASEF). Precursors with m/z between 350 and 1200 were defined in 13 cycles (either 2 or 3 quadrupole switches per cycle) containing 34 ion mobility steps within the ion mobility range of 0.65 – 1.35 (1/k0) with fixed isolation window of 25 Th in each step. The acquisition time of each DIA-PASEF scan was set to 100 ms, which led to a total cycle time of around 1.48 sec. The collision energy was ramped linearly from 65 eV at 1/k0 = 1.6 to 20 eV at 1/k0 = 0.6.

### Spectral deconvolution with DIA-NN

DIA-NN (version 1.8.1) was used to process raw spectra in library-free mode via command line on the Vienna Scientific Cluster (Demichev et al., 2020, 2022). A predicted library search was performed against the human proteome (UP000005640) with match-between-runs (MBR) enabled. Separate MBRs were performed for quality control samples, and ganglia + nerve root, as well as ganglia and nerve root independently when used for differential expression testing. Gene Group outputs are also referred to as “proteins” throughout. See data availability statement for access.

### Basic Gene Lists

Curated lists of pain genes have been previously published (LaCroix-Fralish et al., 2007; Meloto et al., 2018; Zhao et al., 2024). Ion channels were matched from Alexander et al., (2023), and GO-term related gene lists were extracted through R using biomaRt (Durinck et al., 2005). Drug-targets were extracted from opentargets.com for relevant conditions (Ochoa et al., 2023). Receptor types were derived from a previously published interactomics resource (Wangzhou et al., 2021), and were compared to published bulk RNA-seq from the DRG (Ray et al., 2023).

### Gene Set Enrichment Analysis (GSEA)

Gene Set Enrichment Analysis (GSEA) for neuronal subtype enrichment was performed against gene lists derived from Zheng et al., (Zheng et al., 2019) for mouse subpopulations as described previously (Barry et al., 2023). In brief, RNA-seq count data from GSE131230 - which had been processed via STAR alignment and HTSeq on the same genome build (see Zheng et al., for full methods) - was corrected for library size and transformed via rlog in R using DESeq2 (Love et al., 2014; Zhu et al., 2019). This was then filtered to match their published report. Genes with an average rlog above the 95% quantile cut-off per subpopulation were curated into a ‘gene set’ for enrichment. Mouse gene names were converted using the biomaRt package via the ensembl mart with `getLDS()` (Durinck et al., 2005).

For human subpopulations as described in Taveres-Ferreira et al., (Tavares-Ferreira et al., 2022), marker genes were accessed directly from the supplemental tables and reformatted in R for processing. In brief, these genes were selected using the `findMarkers()` function in Seurat after Visium-based spatial sequencing.

These custom gene sets were then compiled for a GSEA analysis using the clusterProfiler package (T. Wu et al., 2021). Minimum gene set size was set to 25 (no maximum size threshold), and run for 10000 permutations.

The average expression across ganglia samples was calculated per gene group after filtering for 80% completeness. Where the first term per gene group overlapped (eg. TRPV1 vs TRPV1,TRPV2,TRPA1), the first term in the group was used, and the row with the higher abundance was taken. GSEA was then performed on the ranked mean expression.

To examine pathway differences between male and female samples, GSEA was performed on ranked LFC output with a significance threshold of FDR < 0.05; minimum gene set size was set to 25. Gene sets were extracted using the `msigdbr` package in R (Dolgalev, 2021). Hallmark pathways were extracted as `category = “H”`. Biological pathways (BP) and molecular function (MF) from gene ontology (GO) lists were extracted as `category = “C5”, subcategory = “BP”` and ` category = “C5”, subcategory = “MF”` respectively.

For the proteomics data, LFC were calculated using limma (see: hypothesis testing). For GSEA on RNA-seq, DESeq2 was used to calculate LFC, as described in Farah et al., (2024), using previously published bulk RNA-seq data (Ray et al., 2023). For ATAC-seq, LFC were derived from pseudobulk analysis of all clusters in a spatial-seq dataset in autosomes, described by Franco-Enzástiga et al., (2024).

### Overrepresentation Analysis

Overrepresentation analysis was performed using the ènrichGÒ function from ClusterProfiler (T. Wu et al., 2021). Differentially expressed proteins (DEPs, abs(LFC) > 1, FDR < 0.05) were compared to a background of Gene Groups used for the initial limma comparison using default settings.

### Supervised PCA (SPCA)

Supervised PCA (SPCA) (Bair et al., 2006) was performed as previously described (Barry et al., 2023). Differentially expressed genes (DEGs) from spatial RNA-seq and differentially expression regions (DARs) from bulk hDRG ATAC-seq datasets exploring sexual dimorphism were extracted as lists (Franco-Enzástiga et al., 2024; Tavares-Ferreira et al., 2022). DARs were filtered to remove X and Y chromosome regions. DEGs from Taveres-Ferreira et al., (2022) were extracted from supplemental table 2, `B-Overall_neurons_DE_genes` looking at differential expression between male and female barcodes within neurons.

Proteomic data from the ganglia (merged by replicate) was subset by either DEGs or DARs and subject to a PCA (via `prcomp` in R). Eigengenes and eigen vectors were then extracted from the first principal component (`PC1`).

### Hypothesis Testing

Hypothesis testing was performed within ganglia samples using a moderated T-Test via limma (Ritchie et al., 2015), modelling for sex. Here, log2 transformed data were first filtered for the primary occurrence of each Gene Group (ie. protein) and filtering out proteins present in less than 80% of samples. For example, where Gene Group A = “TRPV1” and B = “TRPV1,TRPV2,TRPA1”, Gene Group A would be used. P values were corrected with Benjamini-Hochberg, and a significance threshold was set to FDR < 0.05, LFC >1.

### Multi-study Factor Analysis (MSFA)

Strong correlations between ATAC-seq regions, RNA-seq transcript abundance, and protein abundance were not evident. Even so, matching sexually dimorphic pathways are described across datasets. Here, we employed a Multi-Study Factor Analysis (MSFA) (De Vito et al., 2016) to examine latent variables in the context of sex to look for the underlying structure contributing to this overlap.

Following the principles for factor analyses on small sample sizes (de Winter et al., 2009) we limited our search to a small number of factors (here, 3) and used sexually dimorphic DARs. This allows us to look exclusively at chromatin regions implicated in female/male differences: this biases the analysis towards factors involved in sexual dimorphism and provides a clear link from ATAC-RNA-protein. The within-dataset factor numbers were determined by scree plots. A minimum Kaiser–Meyer–Olkin (KMO) threshold was set to 0.5 for each dataset, and only sex-related factors were considered from the output (ie., a confirmatory model, opposed to an exploratory factor analysis). Due to the arbitrary nature of factor loading signs, within-dataset factor loadings were constrained such that they were positively correlated to males if sex appeared relevant.

Bulk RNA-seq from human DRG (Ray et al., 2023) were extracted as quantile-normalized transcripts per million (qnTPM) for neuronal-enriched donor samples (n = 50). Both “pain” and “non-pain” donors were considered here to examine sexual dimorphism across states. Proteomic samples from the “ganglia” and “nerve root” regions of the DRG were used, merged across technical replicates by mean. Here, we acknowledge the limitation that this is a low sample number, and that samples are paired (16 samples from 8 donors). We limited this effect by analyzing only factors shared across two datasets, specifically in the context of sex, and not within the proteomic dataset in isolation.

Genes were matched to bulk DARs from Franco-Enzástiga et al., (2024) and filtered to remove genes with missing values across both datasets, resulting in complete data matrices. Data were then transposed for MSFA (RNA = [50 × 402], proteomics = [16 × 402]). Starting values were extracted using `start_msfà, constraint = `block_lower2`, capped at 10000 iterations. ècm_msfà was then run with default parameters.

Factor scores were estimated using the Weighted Least Squares (ie. the Bartlett Method; equation 1), where X~ is the centred/scaled data matrix for each dataset, i. Lambda are the factor loadings shared across datasets and Psi is a diagonal matrix equal to the specific variances.


$${\hat{f}}_{l}={\left(\Lambda \Lambda^{T}\psi^{-1}\right)}^{-1}\Lambda^{T}\Psi^{-1}({\tilde{X}}_{l})$$

The interaction of `Sex` and `Factor` was tested by an anova. Posthoc testing was then performed per factor (eg. Male.Factor1 - Female.Factor1) using `glht(anova, linfct = mcp(Interaction))` in R. Factors with a q < 0.1 were considered significant. Shared gene loadings were extracted from the MSFA output as Phi for visualization.

GSEA analyses were performed as above, with the following modification: minimum gene set size set to 5 (due to the total number of genes available, 402). In line with above, the significance threshold was set to FDR < 0.05. Protein-protein interaction networks were plotting through https://livedataoxford.shinyapps.io/drg-directory/, (Zhao et al., 2024) which uses an API with STRING DB and overlays sensory neuron and pain-relevant datasets through R Shiny.

### Fluorescent Activated Cell Sorting (FACS) preparation

The DRGs were trimmed of excess connective tissue, fat, and nerve roots to collect the bulb containing neuronal cell bodies. The bulb of each DRG was cut into 3mm sections and placed in 5mL of pre-warmed digestion enzyme containing 2 mg/mL of Stemxyme I (Worthington Biochemical, LS004106), 10ng/mL of recombinant human β-NGF (R&D Systems, 256-GF), and 0.1 mg/mL of DNAse I (Worthington Biochemical, LS002139) in HBSS without calcium and magnesium (Thermo Scientific, 14170–112).

The tubes were placed in a 37° C shaking water bath and triturated every hour until the DRG sections were dissolved (3–4hrs). Samples were filtered through a 70μm mesh strainer and centrifuged at 350g for 5 minutes at room temperature (all subsequent centrifuge steps follow the same parameters). The supernatant was removed, and the pellet was resuspended in a red blood cell lysis buffer (Biolegend, 420301) and incubated at room temperature for 5 minutes. Samples were then centrifuged, the supernatant was removed, and the pellet was resuspended in 0.5% bovine serum albumin in 1X Phosphate Buffered Saline (PBS). To remove myelin from the dissociation, cells were incubated with myelin removal beads (Miltenyi Biotec, 130–096-433) for 15 minutes at room temperature. Cells were washed with 1mL of 0.5% bovine serum albumin in PBS, spun down, resuspended in 0.5% bovine serum albumin in PBS, and passed through a LS column (Miltenyi, 130–042-401) on a MidiMACS separator (130–042-301) according to manufacturer’s protocol. Samples were then spun down and resuspended in PBS and proceeded to cell staining.

Cells were stained with a fixable live/dead stain (Biolegend, 423107) for 10 minutes at room temperature, protected from light. Cells were washed with 1mL of flow cytometry staining buffer (Invitrogen, 00–4222-26), spun down, and resuspended in flow buffer. Cells were incubated with an Fc receptor blocker (TruStain FcX, Biolegend, 422302) for 10 minutes at room temperature, protected from light. Cells were then incubated with CD45, CD11b, and CD3 antibodies for 30 minutes on ice, protected from light (See Supplemental Table 10 for antibodies used in flow cytometry experiments). Cells were washed with 1mL of flow cytometry staining buffer, spun down, and resuspended in flow buffer and kept on ice until processing. Fluorescently activated cell sorting (FACS) was used to isolate Live, CD45+, CD11b+, and CD3− cells on a BD FACSAria Fusion (Gating Strategy in Supplemental Figure 9A). Cells were collected, spun down, and resuspended in pre-warmed RPMI media (Gibco, 11875–093) containing 10% HyClone^™^ Fetal Bovine Serum (Thermo Fisher Scientific, SH3008803IR) and 1% penicillin/streptomycin. Cells were plated at 20k cells per well in a 96 well plate and allowed to acclimate overnight in an incubator (37°C, 5% CO2). All antibodies are listed in Supplemental Table 10.

### LPS Stimulation

Cells were stimulated with lipopolysaccharide (LPS) (10ng/mL, Sigma-Aldrich, L6529) or its vehicle (RPMI media) with Brefeldin A (1:1000, Biolegend, 420601) for 16 hours. Following stimulation cells were collected and fixed with a Cyto-Fast fixation and permeabilization kit (Biolegend, 426803) for 20 minutes at room temperature. Cells were washed twice with the Cyto-Fast perm wash solution and then incubated with a TNFα antibody for 30 minutes on ice. Cells were washed with 1mL of flow cytometry staining buffer, spun down, resuspended in flow buffer, and kept on ice until data acquisition on a BD LSRFortessa. The percentage of cells expressing TNFα were calculated for each condition using FlowJo (Version v10.10) (Gating Strategy in Supplemental Figure 9B).

### TNFα Stimulation

Cells were stimulated with TNFα (10ng/mL, R&D Systems, 210-TA) or its vehicle (1X PBS) for 30 minutes. Cells were then collected and fixed with 4% paraformaldehyde (Electron Microscopy Sciences, 15710) in PBS for 15 minutes at room temperature. Cells were then washed 2x with flow cytometry staining buffer and permeabilized with pre-chilled, 100% methanol (Fisher Scientific, A456–212), for 30 minutes on ice. Cells were then washed 2x with flow cytometry staining buffer and incubated with p38 and p65 antibodies in flow buffer for 30 minutes on ice. Cells were washed with 1mL of flow cytometry staining buffer, spun down, resuspended in flow buffer, and kept on ice until processing. The percentage of cells expressing p38 and p65 along with the median fluorescent intensity of each signal was calculated for each condition (Gating Strategy in Supplemental Figure 9B). The median fluorescent intensity values were normalized to an unstained control.

### Targeted Phosphorylation Array

Phosphorylation levels were measured in remaining hDRG protein samples using the Proteome Profiler Human Phospho-Kinase Array Kit (R&D Systems, CAT# ARY003C). The commercial protocol was followed, with the following changes: each membrane was washed independently using 7 ml Wash Buffer in 60×15 mm culture dishes, and membranes were rinsed 1x with wash buffer after antibody incubation prior to 3×10 min washes. A ChemiDoc MP Imaging Platform (Bio Rad) was used to detect signals. Four samples per condition were processed for membrane “A”, while 3F + 4M were processed for membrane “B”.

Briefly, the protocol is as follows: membranes were blocked with “Array Buffer 1” for 1 hr at RT prior to an overnight incubation at 4°C with 250 ug protein per membrane (ie. 500 ug per membrane pair, A&B), diluted to 1 ml in “Array Buffer 1”. Membranes were then washed (3×10 min), incubated with membrane-specific antibodies for 2 hr at RT, rinsed 1x prior to washing (3×10 min), and incubated with Streptavidin-HRP for 30 min at RT. After washing (3×10 min), membrane were incubated for 90 seconds with the “Chemi Reagent Mix”, blotted with Kimwipes and imaged on a ChemiDoc MP Imaging Platform for 240s. All membranes per round (2M/2F per round) were developed and imaged together.

Image quantification was performed in ImageJ. Regions of Interest (ROIs) of equal size were processed using the ROI Manager, and quantification was considered as ${\overset{¯}{\text{X}}}_{\text{(pixel}\;\text{intensity,}\;\text{PBS)}\;}-{\overset{¯}{\text{X}}}_{\text{(pixel}\;\text{intensity},\;\text{ROI})}$ for each phosphorylation site, using the PBS negative control per membrane as the background.

### Graphics

All plots were generated in R with the following libraries unless otherwise described: ggplot2, ComplexHeatmap, cowplot, gridExtra, ggbiplot, ggrepel. Figures were compiled in Inkscape.

## Supplementary Material

Supplement 1

Supplement 2

Supplement 3

Supplement 4

Supplement 5

Supplement 6

Supplement 7

Supplement 8

Supplement 9

Supplement 10

Supplement 11

Supplement 12

## Figures and Tables

**Table 1.** Donor details.

donor	Sex	Age	DRG	Pain	HTN	Ethnicity	COD

MS-5	F	32	L2	FALSE	FALSE	Black	Head Trauma/GSW
MS-3	F	41	L3	FALSE	TRUE	White	Head Trauma/Motorcycle Accident
MS-7	F	41	L2	FALSE	TRUE	White	CVA/Stroke
MS-2	F	55	L3	FALSE	TRUE	White	Anoxia/Cardiovascular
MS-1	M	33	L3	FALSE	TRUE	White	Head Trauma/MVA
MS-8	M	41	L4	FALSE	TRUE	Black	Head Trauma/GSW/Suicide
MS-4	M	43	L3	FALSE	FALSE	Hispanic	Head Trauma/MVA
MS-6	M	53	L1	FALSE	FALSE	Hispanic	Anoxia/Seizure
FACS-1	M	34	2xL3+T8	NA	NA	NA	Anoxia

MS: mass spectrometry; FACS: fluorescent activated cell sorting; HTN: hypertension, COD: cause of death; GSW: gunshot wound; MVA: motor vehicle accident; CVA: cerebrovascular accident

**Table 2.** Example clinical outcomes on TNFα inhibitors and sexual dimorphism

PMID	Citation	Title	Condition	Relevant outcome

*Primary examples*				
PMID: 19815494	(Van Den Bosch et al., 2010)	Effectiveness of adalimumab in treating patients with active psoriatic arthritis and predictors of good clinical responses for arthritis, skin and nail lesions	PsA	Male sex increased the chance of achieving a good clinical response
PMID: 28762060	(Chimenti et al., 2017)	A 2-year observational study on treatment targets in psoriatic arthritis patients treated with TNF inhibitors	PsA	Females less likely to achieve remission
PMID:16705046	(Hyrich et al., 2006)	Predictors of response to anti-TNF-α therapy among patients with rheumatoid arthritis: results from the British Society for Rheumatology Biologics Register	RA	Females less likely to achieve remission
PMID: 23322995	(Zelinkova et al., 2012)	Sex-dimorphic adverse drug reactions to immune suppressive agents in inflammatory bowel disease	IBD	Higher ADR in women taking TNFα-inhibitor
PMID: 31310690	(Schultheiss et al., 2019)	Earlier discontinuation of TNF-α inhibitor therapy in female patients with inflammatory bowel disease is related to a greater risk of side effects	IBD	Higher ADR in women taking TNFα-inhibitor
PMID:12492735	(Crandall & Mackner, n.d.)	Infusion reactions to infliximab in children and adolescents: frequency, outcome and a predictive model	Crohn’s	Female sex as a predictor for side effects in TNFα-inhibitors for Crohn’s
*Example Reviews*				

PMID: 37820857	(Cignarella et al., 2023)	Sex-oriented perspectives in immunopharmacology	Mixed	TNFis show “higher efficacy and adherence in males, More frequent serious infections in males, more frequent toxic liver disease and lupus-like syndrome in women”
PMID: 29754330	(Rusman et al., 2018)	Gender Differences in Axial Spondyloarthritis: Women Are Not So Lucky	AS	Increased TNF levels in only male AS patients, treatment efficacy of TNFi is significantly lower in women compared to men with axSpA, and they have a significantly lower drug adherence
PMID: 26490106	(Souto et al., 2016)	Rate of discontinuation and drug survival of biologic therapies in rheumatoid arthritis: a systematic review and meta-analysis of drug registries and health care databases	RA	Female sex as a predictor of dicontinuation

PsA: Psoriatic arthritis, RA: Rheumatoid Arthritis, IBD: Irritable Bowel Disease, AS: Axial Spondyloarthritis

**Table 3.** Summary of pain-related GWAS hits for the TNFα signalling pathway from the HPGDB (Meloto et al., 2018)

Loci	Variants	direction	Phenotype	PMID	comments from HPGDB, truncated with [...]

	rs1800629	up	Analgesia	PMID:18990769	AKA TNF-308G/A
	rs1800629	up	Analgesia	PMID:18990769	AKA TNF-308G/A, patients with this genotype did not show improvement in pain scores between two assessment periods
	rs1800629	up	Migraine	PMID:20035431	A borderline association was observed in TNFA 308GA genotype in migraine patients versus controls. [...]
	rs1799724	up	Migraine	PMID:25434717	TNF-α-857 CT genotype and T allele were associated with increased risk of migraine. TNF-α-857 CT genotype was associated with increased risk of migraine without aura (MO) or with aura (MA) in females or males. *While -857T allele was significantly associated with MO or MA in males and with MA only in females*.
	rs1800629	up	Migraine	PMID:25434717	TNF-α-308 GA, AA genotypes and A allele were associated with increased risk of migraine. TNF-α-308 GA, AA genotypes and A allele or AA genotype were associated with increased risk of migraine with aura (MA) and migraine without aura (MO) respectively; *this was more significant in female patients with MA than in males*.
TNF	rs1800610	up	Cancer Pain	PMID:25304131	5-fold increase in the odds of having high pain and high fatigue.
TNF	rs3093664	down	Migraine	PMID:25315199	Associated with increased risk of all migraine types and the subgroup of MRM (Menstrually-Related Migraine).
TNFRSF11B	rs2073618	up	Cancer Pain	PMID:26798969	Increased risk of pain with Aromatase Inhibitor therapy for musculoskeletal toxicity in breast cancer patients.
TNFRSF11B	rs2073618	up	TMD disorders	PMID:30795980	In individuals carrying the CC genotype, there was a 10.80 times greater chance of presenting with TMJ ankylosis. [...]
TNFRSF1A	rs767455	up	Neuraxial pain	PMID:30075559	A higher frequency of G allele was observed in the ankylosing spondylitis group compared with the control group. [...]
TNFRSF1B	rs1061622	up	Cancer Pain	PMID:23852407	Predictive for the symptom cluster of pain, depressed mood and fatigue in lung cancer patients
TNFRSF1B	rs1061622	up	Analgesia	PMID:30075559	Associated with long-term efficacy of etanercept.
NFKBIA	rs8904	down	Cancer Pain	PMID:19773451	Reduced risk of pain in lung cancer patients
TNC	rs1330349	up	Arthritis	PMID:34450027	Significantly associated with hip osteoarthritis
TNC	rs1330349	up	Post-operative pain	PMID:34450027	Significantly associated with total hip replacement pain
TLR2	rs3804100	up	Analgesia	PMID:27649267	This SNP was associated with reduced morphine use overall [...]
ICAM1	rs5498	up	Migraine	PMID:25145994	[...] polymorphism between migraine cases and controls, and between a migraine without aura subtype of migraine cases and control.

TMD: temporal mandibular

## Data Availability

Proteomic data have been deposited via SPARC, DOI:10.26275/z7uy-kuif, and have been added to a searchable database at https://sensoryomics.com/. Corresponding metadata and processed expression matrix are also on github, under `data/processed/*`, and for ease of access the proteomic expression matrix is also included here as Supplemental Table 11. Source data for the targeted phosphorylation array are in Supplemental Table 9; imaged membranes and selected ROIs are shown in Supplemental Figure 10.
